# Interaction of Human Salivary Gland Epithelial Cells with B Lymphocytes: Implications in the Pathogenesis of Sjögren’s Syndrome

**DOI:** 10.31138/mjr.31.4.424

**Published:** 2020-12-28

**Authors:** Efstathia K. Kapsogeorgou, Athanasios G. Tzioufas

**Affiliations:** Department of Pathophysiology, School of Medicine, National and Kapodistrian University of Athens, Greece

**Keywords:** Sjögren’s syndrome, B cells, salivary gland epithelial cells, salivary glands, activation

## Abstract

Sjögren's syndrome (SS) is characterized by the aberrant activation of B-cells in both the target organs of autoimmune responses, such as the exocrine glands and the periphery. Furthermore, SS is strongly associated with the development of B-cell non-Hodgkin lymphomas, which are considered to result from chronic aberrant activation of B-cells. Disturbances of the minor salivary gland (MSG) infiltrating and peripheral B-cells subpopulations have been described in SS patients; however, the underlying mechanisms have not been uncovered. SG epithelial cells (SGECs) play a key role in the development and organization of MSG lymphocytic infiltrates in SS patients. SGECs are suitably equipped to mediate the recruitment, activation, and differentiation of immune cells in SS, including CD4+-T cells. B-cell activating factor (BAFF) secretion by SGECs suggests that they can also fruitfully interact with B-cells and mediate their activation, differentiation, and disturbed subpopulations in SS. The effect of SGECs in the activation and differentiation of naïve peripheral B-cells, as this attested by phenotypical flow cytometric and cytokine production analyses, is under investigation in the current proposal. This approach is expected to enlighten the mechanisms underlying the aberrant activation and differentiation of B cells in SS and the discovery of novel therapeutic targets for its reversal.

## BACKGROUND/INTRODUCTION

Sjögren’s syndrome (SS) is characterized by B-cell hyperactivity in both the target organs of autoimmune responses (exocrine glands, mainly the salivary and lacrimal glands) and the periphery, as attested by profound hypergammaglobulinemia, multiple autoantibodies and cryoglobulinemia.^1^ However, this hyperactivation is not evident in all SS patients. As many other autoimmune diseases, SS is highly heterogeneous with a broad clinical spectrum, extending from mild exocrinopathy (mainly affecting the salivary and lacrimal glands) to severe systemic disease and increased risk to develop B cell non-Hodgkin lymphomas. B cell hyperreactivity is more common in SS patients with immune complex-mediated systemic manifestations, such as purpura, glomerulonephritis, and cryoglobulinemia, whereas it is also associated with extended lymphocytic infiltration of the minor salivary glands (MSG). The heterogeneity of SS clinical manifestations also extends to the autoimmune infiltrates of the MSGs. The grade and composition of the infiltrates, as well as the predominant immune responses, vary among SS patients.^2^ SS patients with mild disease are usually characterised by mild, focal infiltrates around the ducts, whereas T cells (mainly CD4+-T cells) prevail in these infiltrates. On the contrary, patients with systemic disease have been associated with extended diffuse MSG infiltrates leading to loss of tissue architecture. B cells predominate in severe infiltrates, with the later to be quite often organized in ectopic germinal centres (GCs).^2,3^ In addition, these patients often present with features indicative of B cell hyperactivity, such as increased prevalence of various autoantibodies, hypergammaglobulinemia, cryoglobulinemia and immune complex-mediated systemic manifestations, as well as development of lymphoma,^2,4^ suggesting that local MSG and systemic autoimmune responses in SS are linked. In accordance with this, it is generally considered that the chronic incessant activation of B lymphocytes at the MSG autoimmune lesions results in their differentiation and finally transformation and lymphomagenesis. Indeed, MSG lymphocytic lesions are fully blown at diagnosis and remain unchanged in size, composition or predominant immune cell responses over time. The only evolution that has been observed is the development of MALT lymphoma, suggesting that distinct pathogenetic mechanisms operate in patients with severe infiltration of MSGs.^4^ Disturbances of the peripheral B cells subpopulations, as well as of those infiltrating MSGs of SS patients have been described. These include accumulation of memory B cells, transitional-type2 (T2) and marginal zone MZ-like B cells in the MSG lesions, which is accompanied by lower numbers of memory B cells and Bm5 sub-populations, and increased numbers of Bm2/Bm2’ cells in the periphery, compared to control or healthy individuals.^5^ The mechanisms underlying the grade of the MSG infiltrates, the predominant immune cell response (B or T cell), the aberrant activation of B cells, as well as the disturbances of their subpopulations in the MSG autoimmune lesions and the periphery of SS patients are unknown. A plethora of evidence during the last twenty years, support that salivary gland epithelial cells (SGECs) play a key role in the development and organization of lymphocytic infiltrates in the salivary glands of SS patients. Indeed, parallel *in situ* immunohistochemical and *in vitro* studies in cultured SGECs, have shown that they are suitably equipped to mediate the recruitment, activation and differentiation of immune cells in SS,^1^ whereas they have also been shown to mediate the activation and differentiation of CD4+-T cells.^6–8^ Furthermore, SGECs obtained from SS patients have been shown to express functional CD40 molecules and BAFF cytokine,^9,10^ which is essential for B cell survival, activation and differentiation. This suggests that SGECs are possibly capable to fruitfully interact with B cells and mediate their activation and differentiation, as well as the observed disturbances of B cell subpopulations in SS patients.

## AIM OF THE STUDY

The current proposal aims to study the interactions between SGECs from SS patients and naïve B cells from the peripheral blood of healthy donors and particularly the ability of SGECs to mediate the activation and/or differentiation of B cells. The latter is of great importance for the understanding of SS pathogenesis, the uncover of the mechanisms underlying the incessant activation, differentiation and transformation of B cells in SS and the discovery of novel therapeutic targets inhibiting these interactions and/or reversing B cell transformation.

## RESEARCH PLAN – METHODS

The effect of SGECs from SS patients in the activation and differentiation of naïve B cells isolated from the peripheral blood of healthy donors, as this attested by phenotypical analyses and cytokine production will be studied in appropriate co-culture systems that allow either the cell-to-cell contact or the communication only through soluble agents (Transwell co-culture systems). Long-term cultured SGECs will be obtained by a standard protocol developed in our department involving explant outgrowth of epithelial cells from a SG lobule obtained during diagnostic biopsy with informed consent.^11^ The optimal conditions of co-cultures, including the time, need for SGEC fixation, method of B cell isolation and numbers of interacting cells, have already been standardized. B cells were purified by negative magnetic bead isolation from peripheral blood of healthy donors, which uses a mix of antibodies recognizing all the other PBMC populations that are subsequently positively removed by magnetic beads. Thus, the remaining B cells are “untouched” (resting; purity≥98%). The optimal effect of SGECs in B cell activation and differentiation was observed after four days of co-culture (co-culture periods ranging from 3–7 days have been tested), whereas growth arrest by mitomycin or paraformaldehyde chemical fixation of SGECs was not found beneficial for SGEC-B cell interaction. Furthermore, the survival of B cells was not affected as analysed by trypan blue staining. Thus, in the proposed study purified peripheral B cells will be added in confluent cultures of untreated SGEC in 24-well culture plates and will be cultured in RPMI medium which favours B cell culture. In Transwell systems that permit interaction only through soluble factors, SGECs will be cultured to confluency in a 0.4μm pore membrane (upper part) and B cells will be added in the lower part. After 4 days of co-culture, B cells will be collected and their phenotype will be analysed by flow cytometry using specific markers for each B cell subpopulation or indicative of B cell activation, such as CD38, IgD, IgM, CD24, CD21, CD23, CD5, CD27, CD40, CD80, CD86 and BAFF-receptor/CD268. In addition, 48-hrs co-culture supernatants will be collected and analysed by commercially available ELISAs. The effect of SGECs obtained from SS patients in B cells phenotype and cytokine production will be routinely and parallelly compared to this of SGECs obtained from sicca controls, as well as from neoplastic epithelial HeLa cells in all experiments, whereas in certain experiments B cells co-stimulated by CpG will also be tested. The implication of various receptors and/or cytokines that will be implicated in SGEC-B cell interactions will be validated by the addition of specific inhibitory antibodies in co-cultures and subsequent analysis of B cell phenotype and cytokine production. In primary experiments, we analysed the production of several cytokines, including IL1α, IL1β, IL2, IL4, IL5, IL6, IL7, IL8, IL10, IL12, IL13, IL17a, IFNγ, TNFα, TGFβ, G-CSF and GM-CSF, by commercially available qualitative multi-analyte ELISAs. This approach indicated differential expression of IL1α, IL1β, IL6, IL8, IL10, IL12, TNFα, TGFβ, G-CSF and GM-CSF cytokines in co-cultures of B cells with SGECs obtained from SS patients compared to those from sicca-controls or HeLa cells. The differential expression of these cytokines will be confirmed by specific quantitative ELISAs. Furthermore, the SGEC-driven activation/differentiation of B cells will be compared between SGECs derived from SS patients with severe infiltrates and B cell predomination at MSGs and patients with mild infiltration and T cell dominant responses.

All methods and procedures are established in our lab, whereas the proposed study has been approved by the Ethics Committee of School of Medicine, NKUA, Greece (Protocol-No.: 231/28/1/2020).

## IMPACT OF THE STUDY

The study of the effect of SGECs in the activation and differentiation of B lymphocytes is expected to be of high importance for the delineation of the pathogenetic mechanisms underlying the disturbances of B cell subpopulations in SS. As lymphomagenesis in SS is considered the result of the chronic incessant activation of B cells, the proposed study is anticipated to enlighten the SS-related lymphomagenesis, as well. The understanding of the pathogenetic mechanisms underlying the disturbances of B cell subpopulations in SS and the progression to lymphoma is mandatory for their effective treatment and/or reversal, as well as the discovery of novel therapeutic targets.
